# A Novel 110-kDa Receptor Protein is Involved in Endocytic Uptake of Decorin by Human Skin Fibroblasts

**DOI:** 10.1100/tsw.2006.17

**Published:** 2006-01-17

**Authors:** David Denis Sofeu Feugaing, Hans Kresse, Robert R. Greb, Martin Götte

**Affiliations:** ^1^ Department of Obstetrics and Gynecology, Münster University Hospital, Münster, Germany; ^2^ Department of Physiological Chemistry and Pathobiochemistry, Münster University Hospital, Münster, Germany

**Keywords:** glycosaminoglycan, dermatan sulfate, endocytosis receptor, small leucine-rich proteoglycan, biglycan, photoactivated cross-linker

## Abstract

The small leucine-rich proteoglycan (SLRP) decorin is efficiently internalized by a variety of cultured cells. A 51-kDa protein has previously been described as a receptor mediating endocytosis of decorin and of the structurally related SLRP biglycan. Recent findings suggest that endocytosis of SLRPs may also be mediated by additional receptors. The class-A scavenger receptor, the endocytic mannose receptor, the epidermal growth factor receptor, and insulin-like growth factor-I receptor have emerged as candidates. We used a combined approach of immunoprecipitation and photoactivated cross-linking to identify endocytosis receptors for decorin in human skin fibroblasts. Decorin was purified by HPLC-DEAE-ion exchange chromatography from the secretions of human skin fibroblasts under nondenaturing conditions. Confocal immunofluorescence microscopy revealed that both biotinylated decorin and decorin conjugated to the heterobifunctional cross-linker sulfosuccinimidyl 2-(p-azidosalicylamido)ethyl-1-3'-dithiopropionate (SASD) were endocytosed with equal efficiency. SASD-conjugated decorin was added to [^35^S]-methionine-labeled fibroblasts and cross-linked intracellularly to receptor molecules by photoactivation on endocytic uptake. Cross-linked decorin-receptor complexes were purified from the extracts of trypsin-treated fibroblasts by anion exchange chromatography and immunoprecipitation with a decorin-specific antiserum. Analysis by 2D electrophoresis and autoradiography revealed that decorin was specifically cross-linked to a protein of 110 kDa, which exhibited an isoelectric point of 5.5. In a second approach, unlabeled fibroblasts were subjected to decorin endocytosis and photoactivated cross-linking followed by Western blotting of DEAE-purified cell extracts. A shift of biotinylated decorin immunoreactivity from 165 kDa (decorin-receptor complex) to 54 kDa (SASD-conjugated biotinylated decorin) was noted on reductive cleavage of the cross-linker, representing a difference in molecular weight of approximately 110 kDa. The identification of a 110-kDa protein as a novel endocytosis receptor for decorin provides further support for the emerging concept of a redundancy of receptor molecules in the endocytosis of SLRP.

[+]Deceased.
*Corresponding author
©2006 with author.
Published by TheScientificWorld, Ltd. www.thescientificworld.com

### INTRODUCTION

Decorin is a member of the small leucine-rich repeat proteoglycan (SLRP) family[1,2]. A single chondroitin/dermatan sulfate glycosaminoglycan chain is linked to a serine residue at the N-terminal region of its approximate 40-kDa core protein, which can be substituted with up to 3 N-linked oligosaccharides[1,2]. The protein core contains 10 leucine-rich repeats (LRR) of 24 amino acids[1,3]. A recent study on the crystal structure of the decorin core protein revealed that decorin monomers form a curved solenoid fold characteristic of LRR domains, with a parallel ß-sheet on the inside[3]. It was shown that decorin dimerizes through the concave surfaces of the LRR domains[3], which may be a means of facilitating decorin-receptor dimerization processes *in vivo*. The C-terminal region of the dimeric decorin core proteins forms an unusual capping motif involving a laterally extended LRR and a disulfide bond, which is unique to SLRPs[3]. Via its core protein, decorin interacts with several components of the extracellular matrix, including collagen types II, III, and VI, fibronectin, C1q, and transforming growth factor-ß[1,2]. The most noted phenotype of decorin knock-out mice is skin fragility and abnormal collagen fibril morphology, supporting its role in the lateral assembly of collagen fibrils[4]. Moreover, decorin has been implicated in the control of cell proliferation by inducing arrest of tumor cells in the G1 phase of the cell cycle[5]. The interaction of decorin and the epidermal growth factor receptor (EGFR) triggers a signal cascade leading to activation of mitogen-activated protein kinases, mobilization of intracellular calcium, up-regulation of p21WAF1/CIP1 (p21), and ultimately to growth suppression[6,7,8]. In line with these observations, a down-regulation of decorin expression has been observed in human breast carcinomas, endometrial cancer, and ovarian tumors[9,10,11,12]. Moreover, decorin expression has been shown to suppress tumorigenicity in mouse and *in vitro* models[13,14,15]. The extracellular concentration of decorin is determined both by the rate of biosynthesis and of degradation. It has previously been shown that decorin is internalized by receptor-mediated endocytosis followed by targeting to the lysosomal compartment, where degradation occurs[16,17,18,19]. However, while many aspects of decorin metabolism are well investigated, little is known about the mechanisms of its internalization and intracellular trafficking up to lysosomal degradation. A decorin-binding protein of 51 kDa, which is present both at the cell surface and in endosomes, has been previously described as a decorin endocytosis receptor[17,18,20]. The same receptor molecule appears to mediate endocytosis of the SLRP biglycan, which is structurally highly related to decorin[21,22]. However, this receptor displays slow kinetics, and no signaling function could be attributed to this molecule. Based on the differential competitive inhibitory effect of heparin/heparan sulfate oligosaccharides on decorin endocytosis and receptor binding, an involvement of additional receptor molecules has been suggested[23]. Among these, the EGFR, which has been demonstrated to bind decorin[6], has emerged as another candidate molecule for decorin uptake[6,24,25]. This interaction causes down-regulation of EGFR levels and signaling, leading to growth suppression[7,25]. Of note, we could recently show that pharmacological inhibition of EGFR tyrosin phosphorylation results in a marked reduction of decorin endocytosis, but not of biglycan uptake[22,26,27]. In macrophages, the class-A scavenger receptor has been identified as an endocytosis receptor for both decorin and biglycan[28], whereas the endocytic mannose receptor has been shown to bind biglycan[29]. Most recently, a functional interaction of decorin with the insulin-like growth factor-I receptor (IGF-IR) has been demonstrated[30]. Because of this potential redundancy of receptor proteins capable of decorin endocytosis, we aimed at identifying receptor proteins mediating decorin endocytosis in human skin fibroblasts, using an approach of photoactivated intracellular cross-linking combined with immunoprecipitation of decorin-receptor complexes. Using two different experimental setups, we could identify a previously undescribed decorin-receptor protein of 110 kDa, which exhibits an isoelectric point of 5.5. Our findings support the emerging concept of receptor redundancy in decorin endocytosis and pave the way towards a mechanistic understanding of the complex process of decorin endocytosis.

## MATERIALS AND METHODS

### Materials

Media, fetal calf serum (FCS), and tissue culture supplies were from Gibco BRL (Eggenstein, Germany). Unless stated otherwise, all chemicals were from Sigma-Aldrich (Taufkirchen, Germany).

### Cell Culture

Human skin fibroblasts were cultured in modified Eagle's minimum essential medium (MEM) with Earle's salts, 10% FCS (Biochrom, Berlin, Germany), 2 m*M* glutamine, 100 U/ml penicillin, and 0.1 mg/ml streptomycin (PAA, Cölbe, Germany) as previously described[31].

### Metabolic Labeling of Skin Fibroblasts with [$^{35}$S]-Methionine

For metabolic labeling of human skin fibroblasts with [$^{35}$S]-methionine, cells were cultured in methionine-free Waymouth MAB 87/3 medium supplemented with 4% of dialyzed FCS[32]. After 1 h of preincubation, labeling medium containing 100 µCi of [$^{35}$S]-methionine (specific radioactivity, 1.07 mCi/µmol; Amersham, Brauschweig, Germany) was added, and incubation continued for 12 h.

### Purification of Native Decorin by HPLC-DEAE Anion Exchange Chromatography

Decorin was prepared under nondenaturing condition from 1.5 l of conditioned human skin fibroblast media collected after 3–4 days of cell culture. Proteinase inhibitors (100 m*M* ε-amino-N-caproic acid, 10 m*M* EDTA, 10 m*M* N-ethyl-maleimide, 0.05 m*M* benzamidinium chloride) and 0.1% Triton X-100 were added to the medium. The medium was subsequently adjusted to pH 7.4 with HCl and directly applied to a DEAE-Trisacryl M (Serva, Heidelberg, Germany) ion exchange column (100-ml gel bed volume) previously equilibrated with 0.2-µm-filtered DEAE starting buffer (20 m*M* Tris/HCl pH 7.4, 150 m*M* NaCl, 0.1% v/v Triton X-100, protease inhibitors) at 4°C. The sample was applied to the column and the flow rate was adjusted to about 500 µl/min. After loading the sample, the column was washed first with five gel bed volumes of starting buffer, and then with five gel bed volumes of washing buffer (20 m*M* Tris/HCl pH 7.4, 300 m*M* NaCl, 0.1% v/v Triton X-100, protease inhibitors). The bound proteoglycans were eluted with three gel bed volumes of elution buffer (20 m*M* Tris/HCl pH 7.4, 1 *M* NaCl, 0.1% v/v Triton X-100, protease inhibitors). Prior to dialysis, the DEAE-Trisacryl M eluate was first concentrated about two times using Aquacid (JBL, Neuhofen, Germany). The sample was filled into a prewetted dialysis membrane and buried in aquacid powder for 3–4 h at 4°C. The concentrated proteoglycan solution was then dialyzed against TBS at 4°C for up to 24 h. After dialysis, the sample was centrifuged (Beckman ultracentrifuge, LS-70) for 30 min at 30,000 rpm and 4°C prior to HPLC-DEAE chromatography. The DEAE-purified and concentrated proteoglycans were now applied onto a HPLC-DEAE matrix to separate heparan sulfate proteoglycans from decorin using a NaCl concentration gradient. The sulfate groups in heparan sulfate molecules have a density distribution different from that of decorin and elute from the DEAE-matrix at lower salts concentrations than decorin[33]. Buffer A (20 m*M* Tris/HCl pH 7.4, 150 m*M* NaCl) and buffer B (20 m*M* Tris/HCl pH 7.4, 1 *M* NaCl) were used to create a continuous salt concentration gradient over time. Decorin elutes from the HPLC-DEAE matrix at a salt concentration between 0.68–1 *M* NaCl[33]. Eluted molecules were detected UV-spectrophotometrically with a diode array detector. Isolated decorin was dialyzed against PBS or endocytosis medium, respectively, for 24 h at 4°C.

### Biotinylation of Decorin

The biotinylation solution was prepared at the moment of use exactly as described by the manufacturer (Pierce, Rockford, USA). All steps were carried out at 4°C. 1.5 mg of Sulfo-NHS-LC-biotin was dissolved in 100 µl dimethyl formamide (DMF) to prepare a 27 m*M* stock solution. 917 µl of a 0.545 µg/µl decorin solution in PBS was gently mixed with 35.3 µl of Sulfo-NHS-LC-biotin stock solution and incubated on ice overnight in a 4°C room. This mixture represents a ratio of Sulfo-NHS-LC-biotin over decorin protein of about 50:1. To remove free Sulfo-NHS-LC-biotin, the solution was added to a 500-µl DEAE column prepared in a Pasteur pipette and equilibrated with 20 m*M* Tris/HCl pH 7.4 containing 150 m*M* NaCl, 0.1% Triton X-100 and protease inhibitor cocktail (Sigma, Taufkirchen, Germany). The column was washed with the same buffer containing 300 m*M* NaCl and biotinylated decorin was eluted in the same buffer containing 1 *M* NaCl without Triton X-100. 500 µl fractions were collected and 5-µl aliquots were dotted onto a nitrocellulose membrane (Schleicher and Schuell, Dassel, Germany). After blocking of the membrane with 3% skim milk in PBS, the membrane was probed with ExtrAvidin-peroxidase (Sigma) (1:1,500 in 1% BSA/PBS), and washed three times with PBS. Fractions containing biotinlyated decorin were detected by an enhanced chemoluminescence (ECL) reaction (Amersham, Braunschweig, Germany).

### Cross-Linking and Immunoprecipitation of Decorin-receptor Complexes

Sulfosuccinimidyl 2-(p-azidosalicylamido)ethyl-1-3'-dithiopropionate (SASD, Pierce) stock solution was prepared by dissolving 3 mg of the reagent in 200 µl of DMSO. Immediately prior to the experiment, a 0.5-m*M* working solution was prepared by mixing 20 µl of stock solution with 980 µl of PBS. To prepare the biotinylated decorin (btn-DCN)-SASD complex, 300 µl of btn-DCN (0.4 µg/µl) was added to 90 µl of SASD working solution and incubated for 12 h at 4°C on a rocker platform. Because of the photoreactivity of the cross-linker, these and the following experimental steps were carried out under darkroom conditions. The endocytosis medium was prepared by mixing the biotinylated decorin-SASD complex solution (390 µl) with 5 ml of endocytosis medium[18,34]. After removal of the labeling medium, the cell monolayer was briefly washed with 37°C-prewarmed Hanks solution and subsequently incubated at 37°C for 10 min with 37°C-prewarmed uptake medium. Upon incubation, the cell plate was immediately placed on ice and washed two times with cold Hanks solution. Then the plate was first exposed to five camera flashes (Yashica, Kyocera, Japan) and subsequently with long-wave UV light (365 nm) for 10 min. After irradiation, the next steps could be carried out in normal daylight conditions. Cells were detached from the culture dish by incubation for 4–6 min with 4 ml of 37°C-prewarmed trypsin solution. Trypsin was quenched by adding 4 ml of cold Hanks solution to the cell suspension. Cells were then pelleted by centrifugation 5 min at 900 × *g* and 4°C. The cell pellet was extracted on ice with 300 µl of extraction buffer (PBS, 1.5% ß-D-octylglucoside, 4% CHAPS, 1 m*M* PMSF, 1x proteinase inhibitor mix). The extract was thoroughly mixed by pipetting up and down and then ultrasonicated on ice (Ultrasonicator, Vetter, Germany) three times for 15 s. After sonication, the sample was centrifuged 10 min at 15,000 × *g* and 4°C. For immunoprecipitation of decorin-receptor complexes, the supernatant was sequentially treated with protein A-Sepharose (Sigma, Taufkirchen, Germany) coated with IgG from control and specific antidecorin antisera, exactly as previously described[34,35].

### SDS-PAGE, Western Blotting, and 2D Electrophoresis

Immunoprecipitated decorin core proteins and intact decorin were separated by SDS polyacrylamide gelelectrophoresis (PAGE) on 12.5% separation gels exactly as previously described[34]. In the case of metabolically [$^{35}$S]-methionine-labeled samples, SDS-PAGE was followed by fluorography[35]. Briefly, gels were dehydrated in three successive changes of DMSO solution for 30 min each. To enhance the radioactive signal, the gel was subsequently incubated for 16 h in a PPO (2,5-diphenyloxazole)-DMSO solution (17% w/v PPO in pure DMSO). The gel was then rehydrated by incubation in a continuous flowing water bath for 30 min and dried under vacuum between two sheets of cellophane film. The dried gel was exposed to an X-ray film (XOR Eastman Kodak, Rockford, USA) and incubated at –70°C. Unlabeled decorin was detected by silver staining following the procedure published by Oakley et al.[36]. Western blotting was performed exactly as previously described[22]. To detect biotinylated decorin, the membrane was probed using peroxidase-conjugated extravidin, followed by enhanced chemoluminescence detection (Super Signal, Pierce) on Kodak X-Ray films. Two-dimensional (2D) electrophoresis with an immobilized pH gradient was carried on using the ZOOM® IPGRunner™ System (Invitrogen, Karlsruhe, Germany). In the first dimension, isoelectrofocusing (IEF) was performed using an immobilized pH gradient (IPG) on ZOOM® strips. The gel on each ZOOM® strip forms a pH gradient with values ranging from 3 to 10. Separation in the second dimension was performed by SDS-PAGE. After immunoprecipition, pellets were resuspended each in 155 µl of rehydration buffer (8 *M* urea, 2% (w/v) CHAPS, 0.5% (v/v) carrier ampholytes, 0.002% (w/v) bromophenol blue. Freshly prepared 2 *M* DTT (1.75 µl) and 1.3 *M* (21 µl) iodoacetamide were added to the suspension to obtain a final concentration of 20 and 150 m*M*, respectively. After gentle mixing, the suspended pellets were heated for 10 min at 70°C and subsequently centrifuged at 20,000 × *g* for 10 min. The supernatant was loaded into the individual channels of the ZOOM® IPGRunner™ cassette. Into these channels the ZOOM® strips were inserted, followed by 14–16 h incubation at room temperature. The first-dimension IEF was subsequently performed using the following step voltages: 200 V for 20 min, 450 V for 15 min, 750 V for 15 min, 2,000 V for 30 min. The second-dimension SDS-PAGE involved reducing and alkylating the proteins focused on the ZOOM® strip in equilibration buffer, loading the strip onto the second-dimension gel, and performing SDS-PAGE. The strips were incubated on a rotary shaker for 15 min at room temperature in diluted NuPAGE® LDS sample buffer (Invitrogen) containing NuPAGE® sample reducing agent (Invitrogen) and freshly added 50 m*M* DTT. Following reduction, the strips were incubated for a further 15 min under the same conditions in the same buffer containing 125 m*M* iodoacetamide. The equilibrated ZOOM® strip was applied to the second-dimension ZOOM® gels (8 × 8 cm, 1.0-mm thick precast Bis-Tris gel, Invitrogen). NuPAGE® MES [2-(N-morpholino) ethane sulfonic acid] SDS running buffer (Invitrogen) was made 0.5% with respect to agarose and used to perform SDS-PAGE for 40–50 min at a voltage of 200 V. [$^{14}$C]-labeled molecular weight marker (Amersham) was used in the marker lane. After electrophoresis, the gel was processed for autoradiography.

### Chondroitin ABC Lyase Digestion of Glycosaminoglycans

Chondroitin ABC Lyase from *Proteus vulgaris* (EC 4.2.2.4, Seikagaku Kogyo, Tokyo) catalyzes the eliminative cleavage of the ß-(1-4) bonds between N-acetyl-D-galactosamine and glucuronic acid or L-iduronic acid in chondroitin sulfates, chondroitin, chondroitin sulfates/dermatan sulfates in hybrid forms, dermatan, and hyaluronic acids chains. After chloroform-methanol precipitation, the dried sample pellets were dissolved in 50 µl of chondroitinase buffer (50 m*M* Tris/HCl, pH 8, 60 m*M* sodium acetate, 60 m*M* NaCl, 0.01% w/v BSA) under vortexing. 5 µl (0.05 units) of enzyme were added to the solution and the mixture was incubated for 2 h at 37°C. The sample was then dried under vacuum in a speed vac concentrator and subsequently washed two times with methanol. The pellet was air dried under a laminar flow hood and solubilized in SDS sample buffer. A control sample was processed as above without addition of enzyme.

### Endocytosis of Decorin and Confocal Immunofluorescence Microscopy

Cells were plated in 35-mm plastic dishes 1–2 days prior to the experiment and cultured in an atmosphere of 95% air/5% $CO_2$. Cells were washed on ice with cold Hank's balanced salt solution (HBSS). Then, 1

ml of endocytosis medium containing 5.2 µg of biotinylated decorin and 10% FCS was added. The plates were incubated 10 min at 37°C to allow uptake of the biotinylated decorin and the plates were placed immediately on ice and washed with cold decorin-free endocytosis medium. Subsequently, cells were washed once with 1 ml trypsin/EDTA and incubated for 5 min at 37°C with 1 ml trypsin/EDTA. To each well, 2 ml of ice-cold medium containing 10% FCS were added and the cell suspension was centrifuged for 5 min at 1,000 × *g* (4°C). The supernatant was carefully removed and cells were resuspended in 500 µl of Hanks solution. 20 µl of cell suspension were pipetted directly onto polylysine-coated microscope slides. After careful removal of surplus medium with Whatman 3MM paper, the cells were fixed with –20°C cold methanol for 5 min and dried at room temperature (RT) under a laminar flow hood. The sample was washed two times with HBSS and blocked with 2% goat serum in HBSS for 30 min at RT. Following two washes with HBSS, the sample was incubated with Alexa555-Streptavidin (Molecular probes, Leiden, NL) diluted 1:1,600 in HBSS/2% goat serum at RT for 2 h. After three washes with HBSS, the samples were mounted in fluoromount G (Biozol, Eching, Germany) and staining was detected by confocal microscopy. Cells that were not subjected to decorin endocytosis served as a negative control. Laser scanning microscopy was performed with a Leica TCS SL confocal microscope. For a control experiment including the competition of unlabeled decorin with btn-DCN, a modification of the procedure described above was used: The skin fibroblasts were cultured in 24 well plates at a density of 50,000 cells/ml. For the endocytosis experiment, the cells were placed on ice and incubated with or without (control) 75 µg/ml of unlabeled human decorin, respectively, for 30 min. After this preincubation period, 5.2 µg/ml of either btn-DCN or btn-DCN-SASD were added to the cells, and the preincubation was continued on ice for 30 min. Subsequently, cells were washed three times with ice-cold endocytosis medium, and endocytosis medium that had been prewarmed to 37°C was added to the cells. The cells were incubated at 37°C for 10 min, trypsinized, and processed for immunofluorescence microscopy as described above. In order to localize cells that have been subjected to competition with cold decorin, an additional staining with a goat polyclonal antibody directed against the endosomal marker protein EEA1 (Santa Cruz, Santa Cruz, USA, 1:100) was performed, using Alexa488-anti goat IgG (Molecular probes, Leiden, NL, 1:1,600) secondary antibodies.

### RESULTS

#### Purification of Native Decorin from Conditioned Media of Human Skin Fibroblasts

In order to identify putative decorin endocytosis receptors in human skin fibroblasts, we purified decorin from the conditioned media of these cells under nondenaturing conditions by anion exchange chromatography as described above. Following a prepurification step on a high-capacity DEAE-anion exchange column, the 1 *M* NaCl eluate, containing heparan sulfate proteoglycans and decorin, was dialyzed and loaded onto a HPLC-DEAE column. Decorin was separated from heparan sulfate proteoglycans using a continuous NaCl concentration gradient over time. Eluted molecules were detected using UV-spectrophotometer with a diode array detector. Under these experimental conditions, decorin started to elute after 42 min, reaching a sharp peak at 43.38 min, and was completely eluted from the DEAE matrix at about 47 min, corresponding to a NaCl concentration of 1 *M*[33] (Fig. 1, peak B). The total elution volume of decorin was about 6 ml. Because of their weaker net anionic charge, heparan sulfate proteoglycans of fibroblasts were eluted earlier from the matrix between 15 and 35 min (Fig. 1, peak A), which correspond to NaCl concentrations of 0.3 and 0.6 *M*, respectively[33]. Purified decorin was dialyzed against PBS for 24 h before being used for further experiments and controlled for its purity by SDS-PAGE and silver staining (Fig. 2). The glycosaminoglycan chain was enzymatically removed by

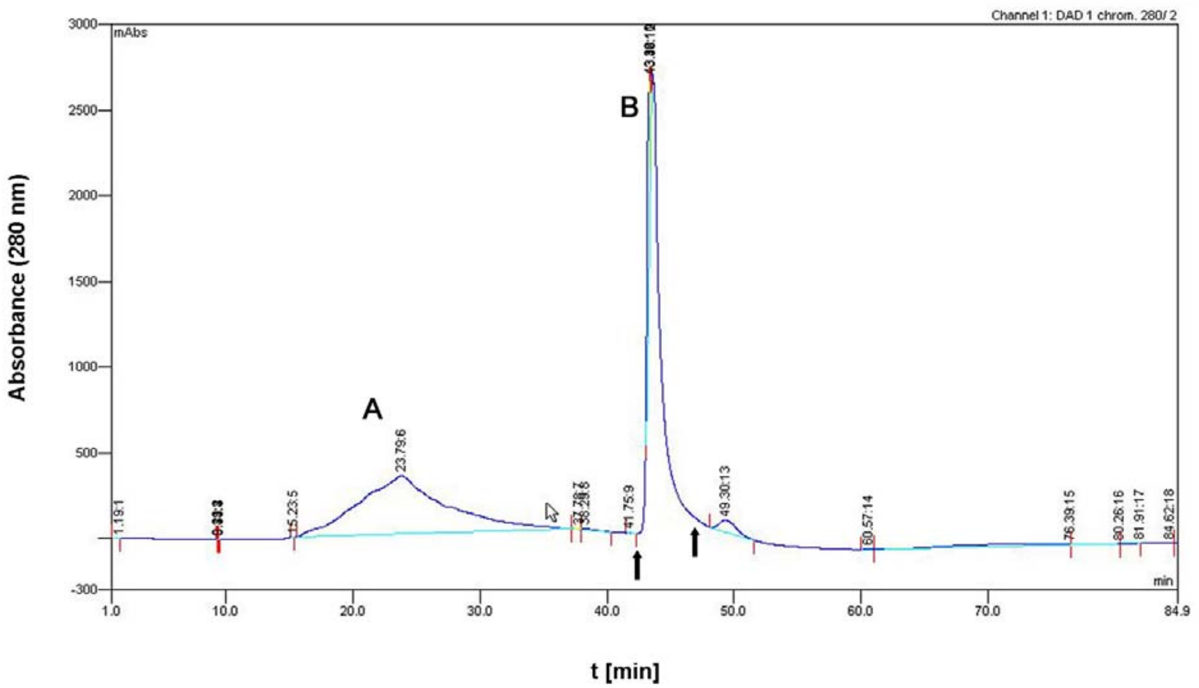

**FIGURE 1.** Purification of decorin from the conditioned media of human skin fibroblasts by DEAE-HPLC chromatography. Media were collected from human skin fibroblast after 3–4 days of culture and prepurified on a DEAE-trisacryl M ion exchange column. After dialysis, decorin was purified by HPLC-DEAE chromatography using a continuous NaCl concentration gradient. Decorin began to elute at about 42 min (B, left arrow) and reached a peak at 43.38 min, whereas heparan sulfate proteoglycans were already eluted between 15 and 35 min (A). Proteins were detected at 280 nm using a diode array detector. Flow rate = 1.2 ml/min. Arrows denote the decorin-containing peak.

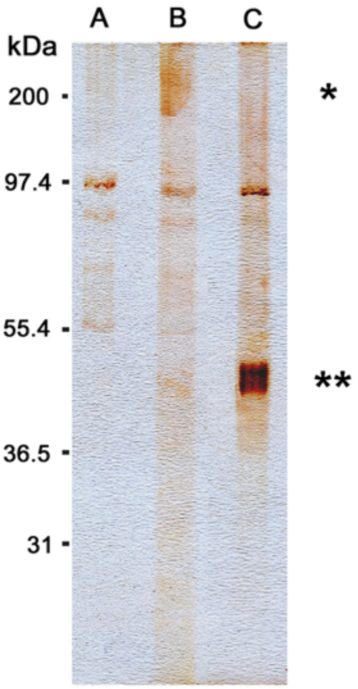

**FIGURE 2**. Silver-stained 12.5% SDS-polyacrylamide gel of decorin samples purified by HPLC-DEAE. Lane A: enyzme control (chondroitinase ABC); lane B: undigested decorin, the position of the proteoglycan form of decorin is indicated by an asterisk (*); lane C: chondroitinase ABC–treated decorin, the position of the core protein of decorin is indicated by a double asterisk (**); left margin: migration positions of molecular weight markers (kDa).

chondroitinase ABC lyase treatment. Undigested decorin was detected as a smear of high molecular weight (around 200 kDa), due to the molecular heterogeneity and size variability of the glycosaminoglycan (GAG) chain (Fig. 2, lane B). When the GAG chain was enzymatically removed by chondroitin ABC lyase treatment (Fig. 2, lane C), the decorin core protein migrated as a more compact sharp band at about 42 kDa. In the negative control lane (digestion buffer and enzyme without decorin substrate), only chondroitin ABC lyase could be detected. The fact that only one additional protein band was detected on lane B or C was taken as proof of the purity of the decorin preparation.

#### Sulfosuccinimidyl 2-(p-Azidosalicylamido)ethyl-1-3'-Dithiopropionate (SASD)–Conjugated Biotinylated Decorin is Internalized by Human Skin Fibroblasts

In order to distinguish between endogenous decorin and exogenously added decorin in endocytosis experiments, we used biotinylated decorin for internalization studies. HPLC-DEAE–purified decorin was biotinylated as described above. After biotinylation, the anionic characteristics of the decorin GAG chain were exploited to separate biotinylated decorin from free biotin, using DEAE-anion exchange chromatography. Eluted biotinylated decorin was detected on a dot blot with peroxidase-conjugated extravidin followed by ECL (not shown) and positive fractions were pooled. Before we started to use decorin in receptor cross-linking studies, our main concern was whether the btn-DCN-SASD conjugate was still endocytosed by human skin fibroblasts. Therefore, we compared endocytic uptake of btn-DCN, which is efficiently internalized by human skin fibroblasts[26,27], and endocytosis of btn-DCN-SASD by confocal immunofluorescence microscopy. btn-DCN was coupled to SASD exactly as described above. Human skin fibroblasts in monolayer culture were incubated for 10 min at 37°C with endocytosis medium[18] containing 5.2 µg/ml of btn-DCN or btn-DCN-SASD, respectively, followed by incubation for a chase period of 10 min with decorin-free endocytosis medium. Cells were trypsinized, fixed, and cytospun onto microscope slides. Internalized decorin was stained using Alexa555-conjugated streptavidin and observed under a Leica TSL confocal laser microscope (Fig. 3). Both btn-DCN- and SASD-conjugated btn-DCN were internalized equally well by the skin fibroblasts. Of note, no cell surface localization of decorin was observed, demonstrating the efficiency of the trypsinization step. To rule out that btn-DCN and btn-DCN-SASD were taken up in a nonspecific manner, an additional control experiment was performed. Skin fibroblasts were preincubated on ice in the presence and absence of nonbiotinylated decorin (75 µg/ml), respectively, for 30 min. Subsequently, 5.2 µg/ml of btn-DCN or btn-DCN-SASD, respectively, were added to the cells and further incubated on ice for 30 min. After washing with ice-cold endocytosis medium, uptake of receptor-bound decorin was induced by incubation in endocytosis medium at 37°C for 10 min. Cells were processed for confocal immunofluorescence microscopy as described above. Under these experimental conditions, both btn-DCN and btn-DCN-SASD were internalized; however, to a lower extent compared to the internalization by cells that were not preincubated at 0°C (Fig. 4A,D). btn-DCN-SASD displayed a slightly increased, more blurry background staining in comparison with btn-DCN, which may be indicative of increased degradation (D.D. Sofeu Feugaing and M. Götte, unpublished) under these experimental conditions. However, both decorin probes were efficiently internalized. Importantly, nonbiotinylated decorin could efficiently compete for the internalization of both btn-DCN and btn-DCN-SASD (Fig. 4 B,E), demonstrating that both modified decorin forms were internalized in a specific manner. Thus, this experimental setup appeared suitable to identify decorin endocytosis receptor proteins.

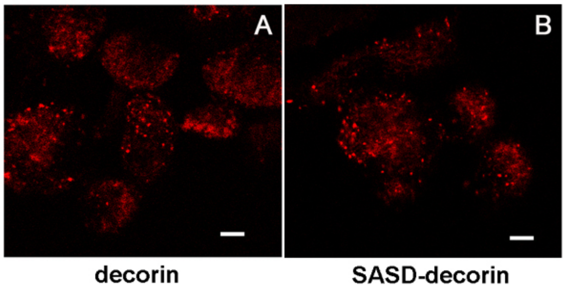

**FIGURE 3.** Uptake of SASD-conjugated biotinylated decorin by human skin fibroblasts. Decorin purified by HPLC-DEAE was biotinylated and conjugated to SASD as described above. Human skin fibroblasts were incubated at 37°C for 10 min with 5.2 µg/ml of btn-DCN (A) or btn-DCN-SASD (B) in endocytosis medium, followed by incubation for a chase period of 10 min with ligand-free medium. Cells were trypsinized and stained for biotinylated decorin using Alexa555-conjugated strepavidin and observed by confocal scanning microscopy. Both btn-DCN and btn-DCN-SASD were internalized equally well. Bars = 6.68 µm.

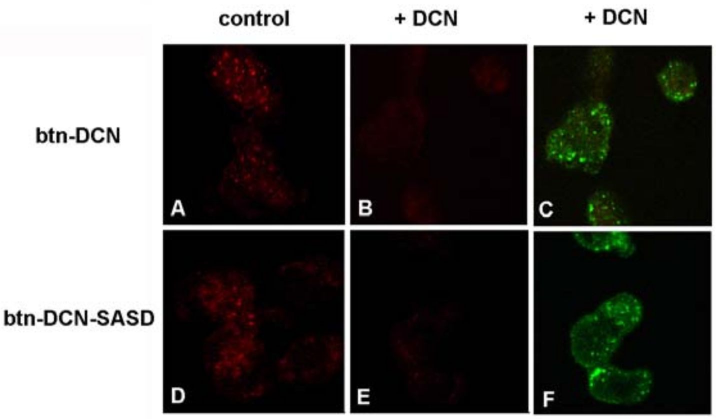

**FIGURE 4.** Internalization of btn-DCN and btn-DCN-SASD is specifically blocked by unlabeled decorin. Decorin purified by HPLC-DEAE was biotinylated and conjugated to SASD as described above. Human skin fibroblasts were incubated on ice for 30 min in the presence (B,C,E,F) or absence (A,D) of 75 µg/ml unlabeled decorin in endocytosis medium, followed by an addition of 5.2 µg/ml of btn-DCN (A-C) or btn-DCN-SASD (D-F) for 30 min. Cells were washed and incubated for 10 min with ligand-free medium at 37°C. Cells were trypsinized and stained for biotinylated decorin using Alexa555-conjugated strepavidin, or for the endosomal protein EEA1 (C,F, green Alexa488 fluorescence) followed by confocal scanning microscopy. Unlabeled decorin efficiently competed for uptake of both btn-DCN and btn-DCN-SASD.

### Photoactivated Intracellular Cross-Linking of Biotinylated Decorin and Putative Endocytosis Receptor Proteins

The experimental strategy for identifying decorin endocytosis receptor proteins was to add SASD-conjugated biotinylated decorin to human skin fibroblasts, to allow the formation of cell-surface decorin-receptor complexes, followed by receptor-decorin complex internalization. Upon endocytosis of decorin, the second reaction of the cross-linker (photoreaction) is triggered to covalently bind decorin to any neighboring molecule. Since decorin has been shown to interact with many proteins of the extracellular matrix and cell surface proteins[6,2,17,18,32], a trypsinization step was employed to remove proteins attached to the cell surface. This allowed us to search exclusively for the internalized cross-linked decorin-receptor complex. Because SASD is a cleavable cross-linker, the protein cross-linked to decorin can be easily separated from the proteoglycan by thiol-ester reduction. This approach appears technically superior to our previous attempts to identify decorin endocytosis receptors that relied on ligand overlay blots[17,18,21] and studies involving cell surface biotinylation and antibody internalization[20,22], respectively. While the previous studies demonstrated the presence of decorin-binding proteins in cell extracts and their presence in endosomes and on the cell surface, respectively, no direct intracellular physical interaction of decorin and its receptor protein(s) was demonstrated.

To identify decorin cross-linker complexes, we used two different experimental approaches. In the first approach, we isolated decorin-receptor complexes from [$^{35}$S]-methionine-labeled fibroblasts subjected to SASD-decorin endocytosis. In this approach, the putative decorin receptors are radiolabeled and can be detected by fluorography after one- or two-dimensional electrophoresis. Specificity for decorin-binding cross-linked proteins is achieved by immunoprecipitation with a decorin-specific antiserum[34].

In the second approach, decorin endocytosis was studied in unlabeled skin fibroblasts. Cross-linked decorin-receptor complexes were purified by anion exchange chromatography and the GAG chains of decorin were enzymatically removed by chondroitin ABC lyase treatment. After SDS-PAGE and Western blotting, decorin and decorin-receptor complexes could be detected by virtue of the biotin tag.

[$^{35}$S]-methionine-labeled skin fibroblasts were subjected to endocytosis of SASD-linked decorin followed by photoactivated cross-linking as described above. Proteoglycans in the cell extract, including the cross-linked decorin-receptor complex, were purified by DEAE-ion exchange chromatography employing a high salt, step-elution protocol. Aliquots of the eluate were subjected to a dot blot, probing the biotin tag with peroxidase-conjugated extravidin, followed by ECL (Fig. 5). Fractions number 1–3, containing the major part of biotin immunoreactivity, were pooled for subsequent immunoprecipitation of decorin.

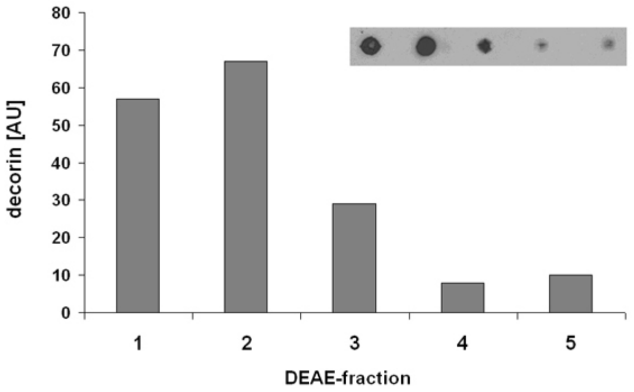

**FIGURE 5.** Fractionated elution profile of cross-linked decorin-receptor complexes subjected to DEAE-anion exchange chromatography. Human skin fibroblasts were incubated for 10 min at 37°C with btn-DCN-SASD. On photoactivation of the cross-linker, cells were immediately trypsinized and extracted with 1.5% ß-D-octylglucoside/4% CHAPS/1 m*M* PMSF/proteinase inhibitors in PBS. Subsequently, proteoglycans were purified from the cell extracts by DEAE-ion exchange chromatography on fractionated elution with the extraction buffer containing 1 *M* NaCl. 20-µl aliquots of the collected fractions were dotted onto a nitrocellulose membrane and biotinylated proteins were detected using peroxidase-conjugated extravidin followed by ECL (insert figure). The dot blot was scanned and analyzed semi-quantitatively using the Image J software (NIH, Bethesda, USA). Fractions 1–3 were pooled for further analysis.

### Immunoprecipitation and SDS-PAGE Analysis of the Decorin-Receptor Complex

Next, decorin cross-linked to putative receptor proteins was immunoprecipitated in the DEAE-purified extract using protein A-sepharose-coupled antibodies as described above. Pooled positive fractions eluted from the DEAE anion exchange column were first incubated with protein A-sepharose-control serum (rabbit preimmune serum). Two rounds of preincubation with freshly prepared protein A-sepharose-control serum preparations were performed. Subsequently, the supernatant was incubated with protein A-

sepharose-rabbit-antidecorin antiserum[34]. Upon incubation, protein A-sepharose beads were thoroughly washed and subsequently boiled in SDS-sample buffer with or without the reducing agent 1,4-dithioerythritol (DTE), respectively, prior to SDS-PAGE. Because the extracted cells were metabolically labeled with [$^{35}$S]-methionine, proteins could be visualized by autoradiography (Fig. 6). Although a stringent washing protocol was applied, a number of proteins of identical molecular weight could be detected both in control-serum and decorin-antiserum precipitates. Since two sequential precipitation rounds of control serum-protein A complexes are used to clear immunoprecipitates[34] in our standard laboratory protocol for immunoprecipitation, and only the second control complex was separated by SDS-PAGE, the control lane shows a weaker signal compared to the decorin immunoprecipitate. Nonetheless, we could observe an enrichment of a specific 113-kDa band in the decorin immunoprecipitate (Fig. 6, arrow). In addition, several additional bands of a higher molecular weight (>205 kDa) could be detected in the decorin immunoprecipitate, suggesting potential redundancy of receptors. This observation prompted us to analyze the immunoprecipitated sample by 2D electrophoresis to obtain a better resolution of proteins specifically enriched in the decorin-antiserum-precipitated sample. Aliquots of the samples separated by SDS-PAGE (Fig. 6) were subjected to IEF and SDS-PAGE in the second dimension as described above. As expected, 2D electrophoresis allowed for an efficient separation of radiolabeled proteins (Fig. 7A). Importantly, as previously observed in conventional SDS-PAGE (Fig. 6), a protein of about 110 kDa that exhibits an isoelectric point of 5.5 could be specifically immunoprecipitated with antidecorin-antiserum (Fig. 7A,B). In contrast, some bands that were present both in preimmune- and decorin-immunoprecipitates showed no differential enrichment and were thus considered nonspecific (Fig. 7B).

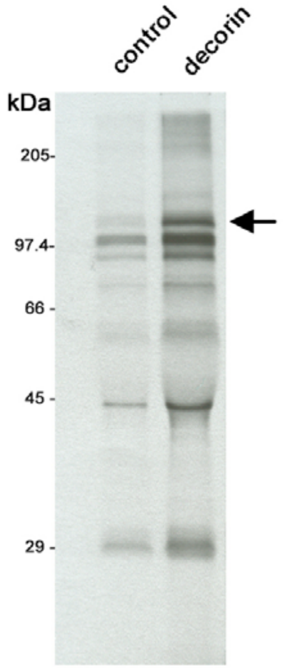

**FIGURE 6**. SDS-PAGE autoradiography of cross-linked decorin-receptor complexes immunoprecipitated from [$^{35}$S]-methionine-labeled fibroblasts. Human skin fibroblasts were steady-state labeled with [$^{35}$S]-methionine and subjected to endocytosis of SASD-conjugated decorin as described in the legend of Fig. 5. Decorin-receptor complexes were immunoprecipitated from DEAE-purified cell extracts. Immunoprecipitates from control preimmune serum (control) and from decorin-antiserum precipitated samples (decorin) were subjected to SDS-PAGE on 12.5% gels under reducing conditions, followed by autoradiography. Only half of the amount of cell extract was used for the preimmune precipitation compared to the precipitation with decorin antiserum, since sequential clearing of the extracts with preimmune sera was performed. The migration position of [$^{14}$C]-methylated molecular weight markers is indicated on the left margin. The arrow denotes the position of a putative receptor protein selectively enriched by decorin immunoprecipitation.

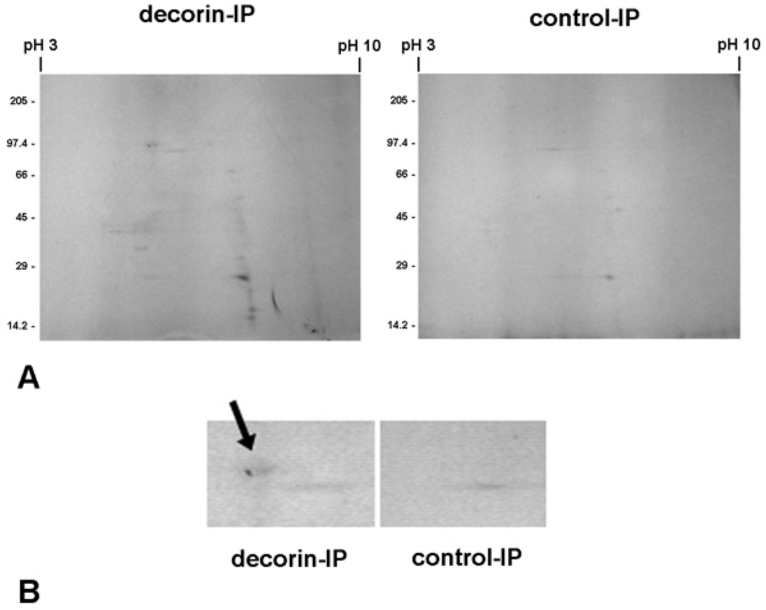

**FIGURE 7.** Separation of cross-linked decorin-receptor complexes by 2D electrophoresis. Cross-linking of intracellular decorin-receptor complexes and immunoprecipitation were performed as described in the legend of Fig. 6. Proteins precipitated with preimmune serum (control) or with decorin antiserum (decorin), respectively, were separated in the first dimension by denaturing IEF in a continuous pH gradient ranging from pH 3–10. Separation in the second dimension was achieved by SDS-PAGE on a 12.5% gel. Proteins were visualized by autoradiography of the dried gels (A). (B) Magnified region from (A): an additional protein band of a molecular mass of about 107 kDa with an isoelectic point of about 5.5 can be detected on the autoradiogram of proteins immunoprecipitated with decorin antiserum compared to preimmune serum.

### Analysis of Cross-Linked Decorin-Receptor Complexes by SDS-PAGE and Western Blotting

We next used a modification of the cross-linking protocol to demonstrate a molecular weight shift of biotinylated decorin immunoreactivity on reductive cleavage of the cross-linked decorin-receptor complexes on Western blots. Unlabeled skin fibroblasts were subjected to endocytosis of btn-DCN-SASD, followed by photoactivated cross-linking as described above. Proteoglycans were purified from the fibroblast extracts by anion exchange chromatography and subsequently treated with chondroitin ABC lyase to remove the dermatan sulfate chains of decorin in order to obtain sharp bands in SDS-PAGE. Aliquots of the purified samples were subjected to SDS-PAGE under reducing and nonreducing conditions, respectively, followed by Western blotting and detection of biotinylated decorin. Under nonreducing conditions, biotin immunoreactivity localizes to a band of approximately 165 kDa (Fig. 8). Although the decorin-receptor complex is easily identified, the signal strength appears weak compared to the overloaded control lanes containing 75 ng of biotinylated decorin. Upon reduction, a clear band shift of biotin immunoreactivity to a position of 54 kDa is observed. The migration position of decorin has shifted towards a higher molecular weight compared with native decorin (cf. Fig. 2) due to the fact that decorin has been biotinylated and modified with the cross-linker (0.54 kDa) that could (theoretically) be linked to several of the approximately 30 lysine residues in the decorin core protein. Since the band shift induced by reductive cleavage of the SASD cross-linker comprises about 110 kDa, this finding confirms the identification of a 110-kDa protein as a receptor for decorin endocytosis. We have previously described a 51-kDa protein as a potential receptor for decorin endocytosis in human skin fibroblasts[17,18,20]. To rule out the possibility that the 51-kDa protein may be a proteolytic fragment of the 110-kDa protein, or that the 110-kDa protein may be a dimer of the 51-kDa protein, we probed a

Western blot of decorin-cross-linked samples with an antibody specific for the 51-kDa protein (Fig. 9). Under reducing

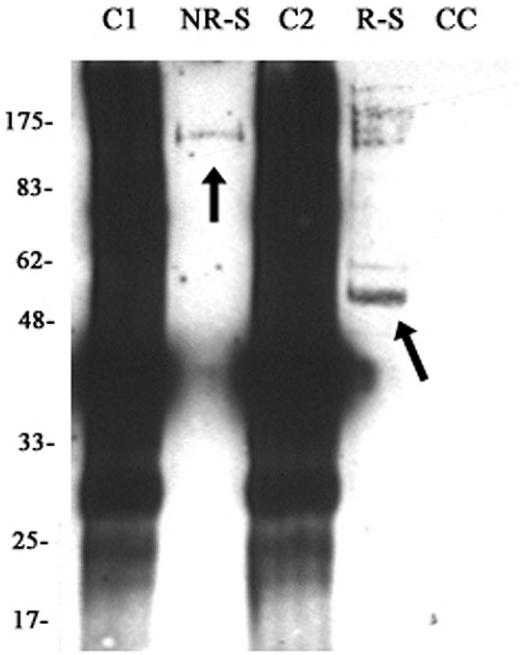

**FIGURE 8.** Western blot of biotinylated decorin core protein cross-linked to the putative endocytosis receptor protein. Human skin fibroblasts in monolayer culture were allowed to internalize btn-DCN-SASD for 10 min, followed by covalent cross-linking of biotinylated decorin to nearby bound proteins upon photoactivation. Cell surface associated proteins were digested by trypsinization at 37°C, cells were extracted, and proteoglycans were purified by DEAE-anion exchange chromatography. Proteoglycans were digested with chondroitin ABC lyase and separated by 12.5% SDS-PAGE under reducing (R-S) and nonreducing (NR-S) conditions, respectively, followed by electrotransfer to a nitrocellulose membrane. As a control, 75 ng of biotinylated decorin (C1, C2), as well as an extract of the same cell line to which no decorin was added, (CC) were used. The application of 75 ng of decorin led to an overloaded signal compared to the DEAE-purified cell extracts. Biotinylated decorin core protein was detected on the nitrocellulose membrane using peroxidase-conjugated extravidin followed by ECL detection. A molecular weight shift of about 110 kDa (arrows) is observed upon release of the decorin core protein from the cross-linked receptor protein by reduction with DTE.

conditions, several bands larger than 51 kDa were observed in addition to the expected 51- and 26-kDa bands. These bands may be nonspecific aggregates or may be indicative of incomplete sample reduction. Importantly, under nonreducing conditions, a prominent band of a molecular mass of 97 kDa could be detected (Fig. 9, asterisk). Since this size is considerably lower than the putative btn-DCN-receptor complex of 165 kDa (cf. Fig. 8), the 110-kDa protein is not immunologically related to the 51-kDa receptor. Thus, the 110-kDa protein appears to be a novel receptor involved in endocytic uptake of decorin by human skin fibroblasts.

### DISCUSSION

The dermatan-sulfate proteoglycan decorin plays important roles in cell adhesion and migration, collagen fibrillogenesis, angiogenesis, and growth factor signaling[1,2,30,39,40]. Of note, defects in the glycosylation of decorin, or changes in its extracellular concentration, have been implicated in the pathogenesis of several diseases, including connective tissue defects, atherosclerosis, and malignant diseases[4,9,10,11,12,13,14,34,41,42]. Since the extracellular concentration is not only determined by its synthesis, but also by its uptake and degradation, it is important to understand the molecular mechanisms of decorin endocytosis. Previously, a protein of 51 kDa has been identified as a receptor for decorin and biglycan endocytosis in a variety of cell types (Table 1). Both endosomal and plasma membrane localization of the 51-kDa protein, which interacts with decorin via its core protein, could be demonstrated[17,18,19,20].

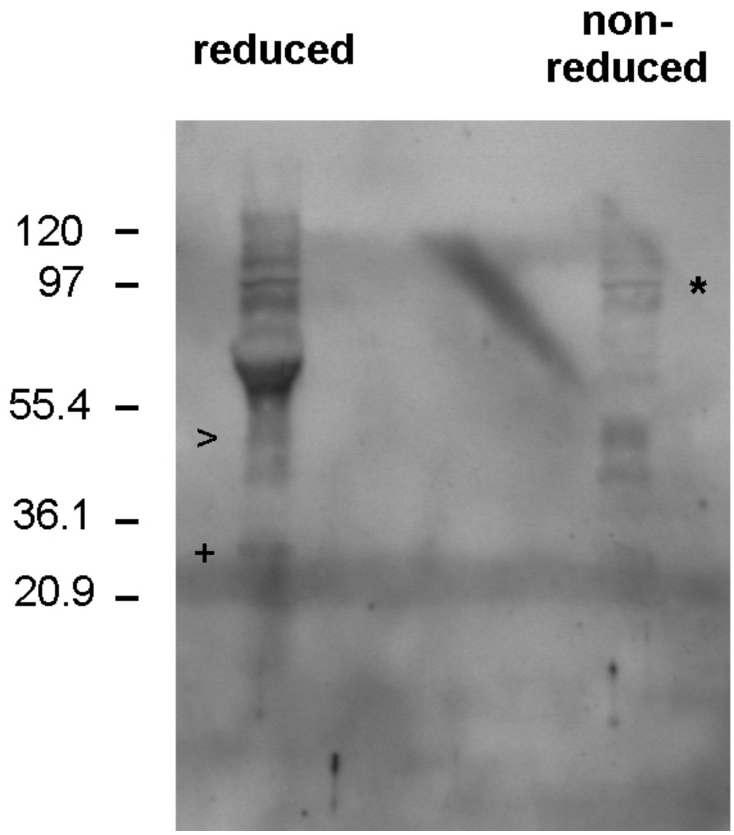

**FIGURE 9.** Western blot of btn-DCN-SASD cross-linked fibroblasts extracts probed for a 51-kDa decorin receptor. Human skin fibroblasts in monolayer culture were allowed to internalize btn-DCN-SASD for 10 min, followed by covalent cross-linking of biotinylated decorin to nearby bound proteins on photoactivation. Samples were processed as indicated in the legend of Fig. 8 and subjected to Western blotting and probing with a monospecific polyclonal antiserum directed against the p51 decorin receptor (1:1,000), followed by incubation with peroxidase-labeled goat-antirabbit IgG antibody and ECL detection[22].Under nonreducing conditions, a protein of a molecular mass of 97 kDa, but not of 165 kDa (cf. Fig. 8), is detected by the p51 antiserum (*). Under reducing conditions, bands of 97 and 60–70 kDa are detected in addition to the 51 kDa (>) and 26 kDa (+) receptor proteins.

However, our own recent findings have shown that decorin appears to utilize several distinct intracellular trafficking routes, based on the differential inhibition of decorin endocytosis by pharmacological inhibitors of selective endocytic routes and on confocal immunofluorescence microscopy colocalization data[20,26,27, D.D. Sofeu Feugaing, and M. Götte, unpublished]. Moreover, several other receptor proteins have emerged as additional candidates for decorin endocytosis (Table 1). Thus, we used an approach of photoactivated intracellular cross-linking combined with immunoprecipitation of decorin-receptor complexes to identify decorin endocytosis receptor proteins in human skin fibroblasts. In contrast to previous approaches, we performed cross-linking of decorin to the receptor *in vivo*, which is technically superior to the ligand overlay blots utilized in previous studies. Using this methodology, we could identify a protein of 110 kDa with an isoelectric point of 5.5 as a receptor for decorin. Several arguments support our view that this protein is an endocytosis receptor for decorin:

1. We could demonstrate that SASD-linked decorin is as efficiently endocytosed as control decorin (Fig. 3).
2. At the time of cross-linking, decorin was internalized (Fig. 3). Only intracellular biotinylated decorin was isolated in the following procedures since extracellular (i.e., membrane and matrix-bound decorin) was effectively removed by trypsinization (Fig. 3).

**TABLE I**
**Putative Decorin/Biglycan Endocytosis Receptors**

| Receptor Protein | SLRP Ligand | Source | kDa | Ref. |
|---|---|---|---|---|
| 110-kDa receptor | DCN | Human skin fibroblasts | 110 | This study |
| 51-kDa receptor | DCN | Human skin fibroblasts, human osteosarcoma cells | 51, 26 * | [18, 33] |
| 51-kDa receptor | DCN | Human keratinocytes | 51, 26* | [37] |
| 51-kDa receptor | BGN | CHO cells, Co60 cancer cells, transformed human breast epithelial cells, human skin fibroblasts | 51, 26* | [22] |
| 51-kDa receptor | BGN | Human skin fibroblasts | 51, 26* | [21] |
| 48-kDa receptor | DCN & BGN | Bovine chondrocytes | 48, 25** | [21] |
| 48-kDa receptor | DCN | Bovine aortic endothelial cells | 48, 25** | [18] |
| Class-A scavenger receptor | DCN & BGN | Murine macrophages | 240 (trimer) | [28] |
| Endocytic mannose receptor | BGN | Human dendritic cells | 175 | [29] |
| EGF receptor | DCN | Human squamous cell carcinoma | 175 | [24] |
| IGF-I receptor | DCN | Human endothelial cells | 135/95 ($\alpha$/ß) | [30] |
| TLR4 (?) | BGN | Murine macrophages | 88 | [38] |
| TLR2 (?) | BGN | Murine macrophages | 86 | [38] |

* 26-kDa protein considered degradation fragment of 51-kDa protein.

** 48,25-kDa proteins considered bovine homologs of human 51,26-kDa proteins.

3. Specificity for the isolation of decorin-receptor complexes was achieved by either using immunoprecipitation with decorin antisera (Figs. 6 and 7) or by detection of biotinylated decorin with streptavidin (Fig. 8). Thus, the cross-linked complex identified by Western blotting via the biotin tag detected only exogenously added decorin, but not endogenous decorin.
4. The band shift observed on reductive cleavage of the cross-linker in the Western blot approach encompasses a similar molecular weight as the receptor protein detected by 2D electrophoresis in the immunoprecipitation approach.

We do not exactly know why the previously described 51-kDa protein was not isolated in this study. By Western blotting, we could demonstrate that the 110-kDa protein is not immunologically related to the 51-kDa protein (Fig. 9), ruling out the possibility that the 51-kDa protein is a proteolytic fragment of the 110-kDa protein, as was previously suggested with regard to the 51- and 26-kDa receptor proteins[37]. Although proteins with a molecular weight of about 50 kDa could be detected by 2D electrophoresis and autoradiography (Fig. 7), these proteins were also present in the control immunoprecipitation (IP) and were thus considered not specific for decorin binding. Furthermore, a band of 26 kDa, which appeared to be stronger in the decorin IP compared to controls, was nonetheless also present in the control IP, whereas the 110-kDa protein was exclusively present in the decorin IP. With respect to the 51-kDa protein, it has previously been shown that this protein is located at the cell surface and in endosomes, and that this protein binds decorin in ligand-blotting assays[17,18,20]. However, the experimental approach used in the present study involved *in vivo* intracellular cross-linking under native conditions and subsequent detection by SDS-PAGE and Western blotting. Thus, we consider our approach technically superior to previous attempts to isolate decorin endocytosis receptors. It is possible that the 51-kDa protein may have copurified with decorin and formed dimers under nonreducing conditions (Fig. 9), however, it may not have been directly cross-linked to decorin upon intracellular photoactivation. Since decorin dissociates from the 51-kDa receptor protein under acidic conditions[17], a major part of internalized decorin may have already been dissociated from the receptor when it has reached acidified endosomal compartments. This pool of potential p51-btn-DCN-SASD complexes would escape photoactivated intracellular cross-linking, whereas the potentially cross-linked pool at the cell surface would be removed by the trypsinization step included in our protocol. We believe that we have isolated a novel receptor for decorin endocytosis, as the molecular weight of 110 kDa does not match the molecular weight of other candidate receptor proteins (Table 1). Both the EGFR (175 kDa) and IGF-IR (135 kDa alpha subunit/95kDa beta subunit) are known to be efficiently endocytosed and to bind decorin[24,30]. In macrophages, the class-A scavenger receptor (80-kDa monomer/240-kDa trimer) has been demonstrated to mediate endocytosis of both decorin and biglycan[28], and biglycan uptake may also be mediated by the mannose receptor (174 kDa)[29]. We have recently shown that in macrophages the Toll-like receptors TLR4 (88 kDa) and TLR2 (86 kDa) also bind biglycan[38]. However, although these receptors have been implicated in the process of phagocytosis[43], very little is known about the endocytosis of Toll-like receptors in general, in particular if these receptors mediate biglycan endocytosis. Although some of these SLRP-receptor interactions are likely to be cell-type specific, they indicate that a certain redundancy in receptors capable of mediating SLRP endocytosis exists. These findings are in accordance with our observation that decorin, and possibly biglycan, utilizes several distinct endocytic pathways[22,26,27]. Our initial cross-linking results indicated the presence of further, high molecular weight potential receptor proteins (Fig. 6), some of which may be identical to receptors listed in Table 1. Thus, the identification of the novel 110-kDa receptor protein supports the emerging concept or receptor redundancy in the endocytosis of SLRPs, which is a further step towards a mechanistic understanding of this complex process.

### ACKNOWLEDGMENTS

This project was financially supported by the Deutsche Forschungsgemeinschaft SFB 492 project A6 (H.K., M.G.) and by an award of the American Society for Cell Biology (D.D.S.F.). The authors would like to thank Petra Blumberg, Monika Offers, Ruth Goez, and Siegmund Budny for excellent technical assistance, and the late Elke Schönherr for discussions.
